# Modulation of the Epithelial-Immune Cell Crosstalk and Related Galectin Secretion by DP3-5 Galacto-Oligosaccharides and β-3′Galactosyllactose

**DOI:** 10.3390/biom12030384

**Published:** 2022-02-28

**Authors:** Veronica Ayechu-Muruzabal, Melanie van de Kaa, Reshmi Mukherjee, Johan Garssen, Bernd Stahl, Roland J. Pieters, Belinda van’t Land, Aletta D. Kraneveld, Linette E. M. Willemsen

**Affiliations:** 1 Division of Pharmacology, Utrecht Institute for Pharmaceutical Sciences (UIPS), Utrecht University, 3584 CG Utrecht, The Netherlands; v.ayechumuruzabal@uu.nl (V.A.-M.); mel_kaa@live.nl (M.v.d.K.); j.garssen@uu.nl (J.G.); a.d.kraneveld@uu.nl (A.D.K.); 2 Department of Chemical Biology and Drug Discovery, Utrecht Institute for Pharmaceutical Sciences (UIPS), Utrecht University, 3584 CG Utrecht, The Netherlands; reshmi.mukherjee@gmail.com (R.M.); bernd.stahl@danone.com (B.S.); r.j.pieters@uu.nl (R.J.P.); 3 Danone Nutricia Research, 3584 CT Utrecht, The Netherlands; belinda.vantland@danone.com; 4 Center for Translational Immunology, The Wilhelmina Children’s Hospital, University Medical Center Utrecht, 3584 EA Utrecht, The Netherlands

**Keywords:** galacto-oligosaccharides, galectins, intestinal epithelial cells, β-3′galactosyllactose, immunomodulation, mucosal immunity

## Abstract

Prebiotic galacto-oligosaccharides (GOS) were shown to support mucosal immune development by enhancing regulatory-type Th1 immune polarization induced by synthetic CpG oligodeoxynucleotides (TLR9 agonist mimicking a bacterial DNA trigger). Epithelial-derived galectin-9 was associated with these immunomodulatory effects. We aimed to identify the most active fractions within GOS based on the degree of polymerization (DP), and to study the immunomodulatory capacities of DP3-sized β-3′galactosyllactose (β-3′GL) using a transwell co-culture model of human intestinal epithelial cells (IEC) and activated peripheral blood mononuclear cells (PBMC). IEC were apically exposed to different DP fractions of GOS or β-3′GL in the presence of CpG, and basolaterally co-cultured with αCD3/CD28-activated PBMC, washed, and incubated in fresh medium for IEC-derived galectin analysis. Only DP3-5 in the presence of CpG enhanced galectin-9 secretion. DP3-sized β-3′GL promoted a regulatory-type Th1 response by increasing IFNγ and IL-10 or galectin-9 concentrations as compared to CpG alone. In addition, IEC-derived galectin-3, -4, and -9 secretion was increased by β-3′GL when combined with CpG. Therefore, the GOS DP3-5 and most effectively DP3-sized β-3′GL supported the immunomodulatory properties induced by CpG by enhancing epithelial-derived galectin secretion, which, in turn, could support mucosal immunity.

## 1. Introduction

Non-digestible oligosaccharides (NDO) are the third major component in human milk [1]. Based on the amount and structure diversity of NDO in human milk, a 9:1 mixture of short-chain galacto- and long-chain fructo-oligosaccharides (GOS/FOS) was studied for its effects on the microbiota and the intestinal mucosa [2]. Various clinical studies have shown that this GOS/FOS mixture promoted the growth of commensal bacteria, induced stool softening, reduced the incidence of infections and the incidence of atopic dermatitis, as well as modulated the antibody profile in infants at high risk of allergy [3,4,5,6,7,8]. Furthermore, when combined with *Bifidobacterium breve* M-16V, it effectively lowered allergic symptoms in a murine model for cow’s milk or hen’s egg allergy in association with increased intestinal and/or serum galectin-9 levels [9,10,11,12]. In addition, in children affected with atopic dermatitis, this synbiotic mixture was found to enhance serum galectin-9 levels after 12 weeks of intervention, in association with reduced atopic dermatitis symptom scores and lower risks of developing asthma [10,13,14].

The major component of the GOS/FOS (9:1) mixture is GOS, which is composed of galactose units coupled to a terminal glucose with a degree of polymerization (DP) ranging between 2 and 8 [13,14]. Upon ingestion, GOS reach the lower parts of the gastrointestinal tract intact where fermentation by the gut microbiota occurs. Consumption of GOS promotes the growth of beneficial commensal bacteria and provides health benefits to the host [15,16]. Besides the microbiota-dependent effects, GOS was also shown to have direct effects on the epithelial barrier. Regarding the effect of GOS on intestinal epithelial cells (IEC), it was shown that GOS can inhibit the adherence of pathogenic bacteria to IEC [17,18], enhance the barrier function by preventing the disruption of gut barrier integrity [19], and promote goblet cell function [20]. Furthermore, GOS supported the absorption of minerals such as iron and calcium in young infants [21,22], as well as lowered the incidence and severity of travelers’ diarrhea in humans travelling to high-risk countries [23].

In addition to the effects on the microbiota and the IEC, NDO such as GOS have been shown to promote direct immunomodulatory effects. GOS was shown to interact with T-cells and dendritic cells, and to selectively promote the release of regulatory IL-10 in vitro [24,25]. The increased IL-10 was also observed in a study performed in suckling piglets [26]. Furthermore, in a double-blind, placebo-controlled study performed in healthy elderly, GOS supplementation positively influenced immune parameters by increasing the production of IL-10, increasing NK cell activity, and reducing pro-inflammatory IL-6 and IL-1β measured in peripheral blood mononuclear cells (PBMC) [27,28].

Although previous studies have shown effects of GOS either on epithelial or on immune cells, we have identified immunomodulatory properties of the NDO mixture GOS/FOS in a transwell model developed to study the crosstalk between IECs and immune cells [29,30,31,32]. The crosstalk between IEC and underlying immune cells is key to maintain the intestinal mucosal homeostasis and to develop appropriate immune responses [33]. Previous studies using this well-established in vitro IEC/PBMC co-culture model reported regulatory-type Th1 responses upon exposure to NDO in association with bacterial DNA or synthetic CpG oligodeoxynucleotides, known to be TLR9 ligands. The immunomodulatory effects observed upon exposure to NDO and CpG in the IEC/PBMC model could support mucosal immune development and were shown to be mediated by epithelial-derived galectin-9, which was found to be a key mediator contributing to the effects observed in vitro [29,30,31,34,35]. These studies also showed that the immunomodulatory properties of CpG and NDO occurred only when the PBMC underlying the IEC were activated, mimicking inflammatory conditions [29].

GOS as the main component in the GOS/FOS mixture contains multiple oligomers (DP2-8), out of which the active immunomodulatory component has not yet been identified. Due to the variety of structures present in GOS, this study aimed to investigate the most active oligomers within GOS by investigating their immunomodulatory capacity using a transwell co-culture model combining IEC and activated PBMC. Therefore, specific GOS DP fractions were isolated by size-exclusion chromatography and exposed to IEC in the presence of CpG oligodeoxynucleotides to study the crosstalk with the underlying immune cells and their effect on IEC-derived galectins. Additionally, we studied the immunomodulatory effects of a specific NDO present in the GOS mixture and found in human milk, namely β-3′galactosyllactose (β-3′GL) [14,36], using the IEC/PBMC co-culture model. Studying the immunomodulatory properties of specific NDO structures will provide further insights regarding their potential role in mucosal immune development.

## 2. Materials and Methods

### 2.1. GOS DP Separation by Size Exclusion Chromatography

**Figure 1:**
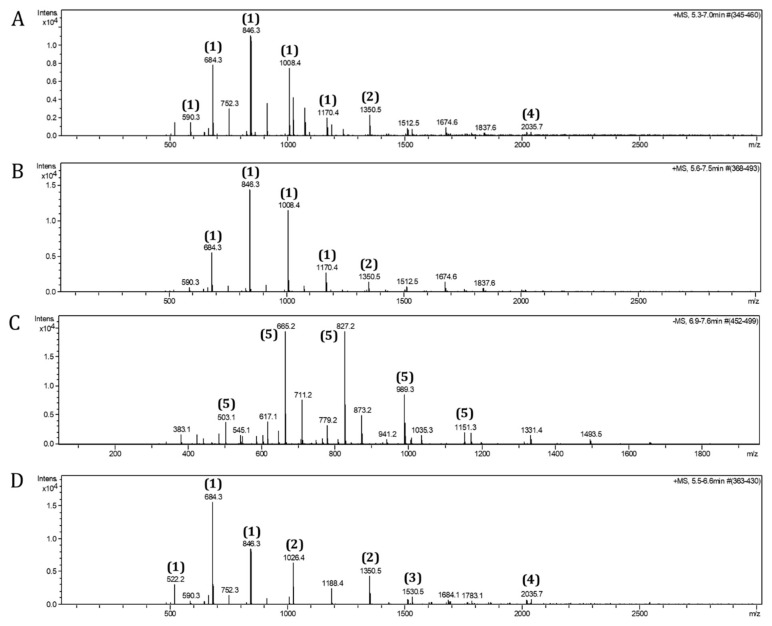
Electrospray ionization-mass spectrometry (ESI-MS) spectra of DP fractions. GOS DP oligomers were separated by size-exclusion chromatography from GOS mixture. The profiles of GOS (**A**), GOS DP4-7 (**B**), GOS DP3-7 (**C**), and GOS DP3-5 (**D**) are shown as ammonia adducts (1) = [M+NH_4_]^+^, (2) = [2M+NH_4_]^+^, (3) = [3M+NH_4_]^+^, (4) = [M+NH_4_]^+^, and (5) = [M, no ammonia adduct].

**Figure 2:**
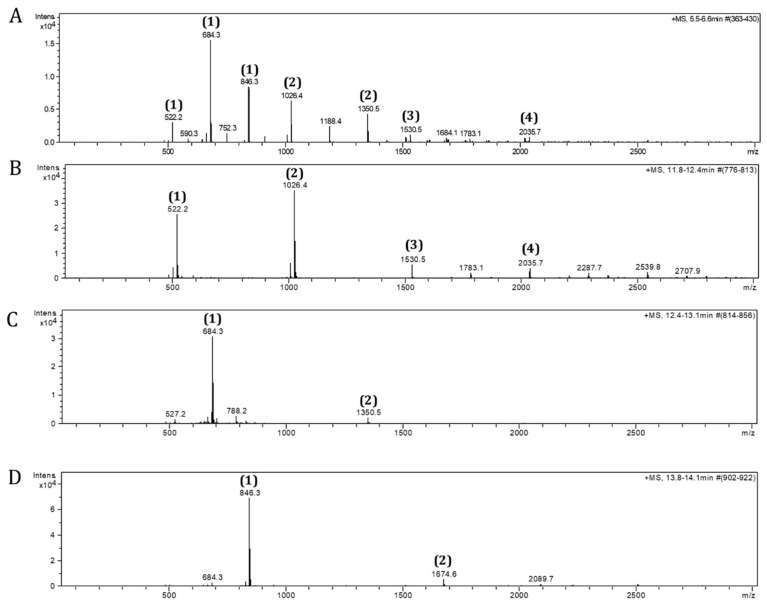
Electrospray ionization-mass spectrometry (ESI-MS) spectra of DP fractions. GOS DP oligomers were separated by size-exclusion chromatography from GOS mixture. The profiles of GOS DP3-5 (**A**), GOS DP3 (**B**), GOS DP4 (**C**), and GOS DP5 (**D**) are shown as ammonia adducts (1) = [M+NH_4_]^+^, (2) = [2M+NH_4_]^+^, (3) = [3M+NH_4_]^+^, and (4) = [M+NH_4_]^+^.

Vivinal GOS syrup (derived from lactose, 45% pure) produced by the elongation of galactose catalyzed by β-galactosidases (Friesland Campina, Amersfoort, The Netherlands) was diluted in Milli-Q water (1:1) and fractionated using a Bio-Gel P-2 column. Milli-Q water was used as eluent. The flow rate used was 0.2 mL/min. The fractions collected (6–12 mL) were freeze-dried (Christ, Osterode am Harz, Germany) and fractions containing DP4-7, DP3-7, or DP3-5 were pooled upon analysis by electrospray ionization-mass spectrometry (ESI-MS) (microTOF-Q-II Bruker, Billerica, MA, USA) using a HILIC column (X-Bridge^TM^ HILIC, Waters, Milford, MA, USA). An amount of 0.1% ammonia was used in the running buffer (acetonitrile:water; gradient of acetonitrile going from 5% to 50% aqueous solution in 10 min) (Figure 1). Additionally, the DP3-5 fraction was further separated into DP3, DP4, and DP5 (Figure 2) using the same experimental conditions as described above.

### 2.2. Culture of IEC

The human colon adenocarcinoma HT-29 cell line (ATCC, HTB-38, Manassas, VA, USA) was used as a model for IEC. The HT-29 cell line was cultured in McCoy 5A medium (Gibco, Invitrogen, Carlsbad, CA, USA) supplemented with 10% fetal calf serum (FCS), penicillin (100 U/mL), and streptomycin (100 µg/mL) (Sigma-Aldrich, St. Louis, MO, USA). IEC were grown in 75 cm^2^ flasks (Greiner Bio-One, Alphen aan den Rijn, The Netherlands) and maintained at 37 °C, 5% CO_2_. Medium was refreshed every 2–3 days. One week before the experiments, IEC were diluted 8–10 times based on surface area and seeded in 12-well transwell inserts (Costar Corning Incorporated, New York, NY, USA). When confluency was reached, the IEC monolayers were used to perform co-culture experiments.

### 2.3. Isolation of PBMC

Buffy coats from healthy donors were purchased (Blood bank, Amsterdam, The Netherlands) and used to isolate human PBMC. Buffy coats were diluted (1:1) using PBS supplemented with 2% FCS. PBMC were isolated by density gradient centrifugation (1000× *g*, 13 min) using Leucosep™ tubes (20 mL per tube) (Greiner Bio-One). After washing, the remaining red blood cells were lysed (4.14 g of NH_4_Cl, 0.5 g of KHCO_3_, 18.6 mg of Na_2_EDTA in 500 mL of demineralized water, sterile-filtered, pH = 7.4). Isolated PBMC were resuspended in RPMI 1640 supplemented with 2.5% FCS, penicillin (100 U/mL), and streptomycin (100 µg/mL).

### 2.4. IEC/PBMC Co-Culture Model

IEC grown in transwell filters were apically exposed to 0.1 or 0.5% (*w*/*v*) GOS DP fractions or β-3′GL (Carbosynth, Berkshire, UK) in the presence or absence of synthetic CpG oligodeoxynucleotides (ODN M362, 0.1, 0.5, or 5.0 µM) (Invivogen, San Diego, CA, USA). In the basolateral compartment, αCD3 and αCD28-activated PBMC were added (2 × 10^6^ cells/mL) (0.15 µg/mL and 0.2 µg/mL, respectively, from Sanquin or BD Biosciences, San Jose, CA, USA) and incubated for 24 h (37 °C, 5% CO_2_), after which the basolateral supernatant was collected and stored at −20 °C for cytokine analysis.

Subsequent to IEC/PBMC co-culture, transwell inserts containing IEC monolayers were collected for quantitative Polymerase Chain Reaction (qPCR) analysis or transferred into a new plate separated from the PBMC and washed with PBS. Then, fresh medium was added and IEC were incubated in fresh medium for an additional 24 h (total 48 h; 24 h in IEC/PBMC co-culture and 24 h in culture of IEC in fresh medium) to determine the IEC-derived basolateral mediator release. In addition, IEC were collected and stored for qPCR analysis.

### 2.5. Enzyme-Linked Immunosorbent Assay (ELISA)

The basolateral supernatants were used to analyze the cytokine and galectin-9 secretion in the IEC/PBMC co-culture, as well as the IEC-derived galectin-3, -4, and -9. Commercially available kits were used to determine IFNγ, TNFα, IL-13 (all from Thermo Fischer Scientific, Waltham, MA, USA), IL-10 (U-Cytech, Utrecht, The Netherlands), and galectin-3 (R&D systems, Minneapolis, MN, USA) following the manufacturer’s protocol. Human galectin-4 and galectin-9 were measured using antibody pairs (both from R&D), as described before [29].

### 2.6. Gene Expression Analysis by qPCR

RNA was isolated from IEC samples using the Nucleospin^®^ RNA Plus kit (Macherey-Nagel, Düren, Germany). Contaminating DNA was removed by incubating with DNAse for 15 min on ice (Qiagen, Hilden, Germany). Complementary DNA (cDNA) was obtained using the iScript™ cDNA synthesis kit (Bio-Rad, Veenendaal, The Netherlands) following the manufacturer’s protocol. The IQ SYBR Green Supermix and CFX96 real-time PCR detection system (both from Bio-Rad) were used for the quantification of gene expression. Commercially available primers for galectin-3, -4, and -9 were used and compared to *RPS13*, as a reference gene (all from Qiagen). Relative mRNA expression was calculated as 100 × 2^(Ct reference − Ct gene of interest)^ [37].

### 2.7. Statistical Analysis

All statistical analyses were performed using GraphPad Prism 8 software (GraphPad Software, San Diego, CA, USA). When the data did not fit a normal distribution, transformation was applied prior to ANOVA analysis. One-way repeated measures ANOVA followed by Bonferroni’s post hoc test with selected pairs were used for the statistical analysis. The conditions with and without CpG were analyzed separately as represented by the dotted line. Within the analysis of CpG-exposed conditions, a comparison between the medium control group and CpG alone was included. Probability values of *p* < 0.05 were considered significant.

## 3. Results

### 3.1. Immunomodulatory Effects of GOS DP Fractions in IEC/PBMC Co-Culture Model

The immunomodulatory effects of DP fractions isolated from GOS were studied using a transwell IEC/PBMC co-culture model used to investigate the crosstalk between epithelial cells and innate, as well as adaptive, immune cells. IEC were apically exposed to the GOS DP fractions DP4-7, DP3-7, and DP3-5 (0.5% *w*/*v*) in combination with 5 µM CpG. In the basolateral compartment, αCD3/CD28-activated PBMC were added and incubated for 24 h.

**Figure 3:**
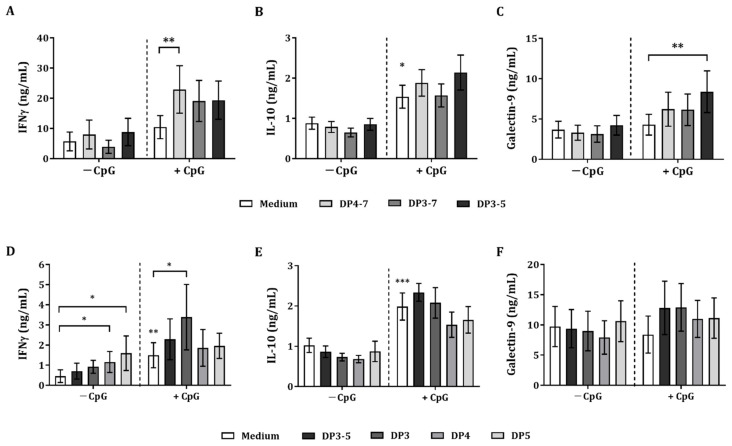
Cytokine and galectin-9 secretion in IEC/PBMC co-culture. IECs were apically exposed to GOS DP4-7, DP3-7, and DP3-5 in combination with 5 µM CpG and basolaterally to αCD3/CD28-activated PBMC. Additionally, GOS DP3-5 was further separated into DP3, DP4, and DP5 fractions. IEC were apically exposed to GOS DP3, DP4, and DP5 in combination with 5 µM CpG and basolaterally to αCD3/CD28-activated PBMC. After 24 h of incubation, IFNγ (**A**,**D**), IL-10 (**B**,**E**), and galectin-9 (**C**,**F**) concentrations were measured in the basolateral supernatant. Data represent mean ± SEM of *n* = 6 independent PBMC donors (except for *n* = 4 in (**A**) and *n* = 5 in (**C**,**D**)). Statistical analysis was performed separately for conditions with and without CpG (represented as dotted line). However, a comparison between the medium control and CpG-exposed condition was included (* *p* < 0.05, ** *p* < 0.01, *** *p* < 0.001).

Exposure of GOS DP4-7, DP3-7, or DP3-5 alone did not affect IFNγ, IL-10, or galectin-9 secretion, but CpG alone enhanced IL-10 secretion by activated PBMC in the IEC/PBMC co-culture (Figure 3A–C). Only combined exposure to GOS DP4-7 and CpG resulted in significantly increased IFNγ concentrations as compared to CpG alone (for DP3-7 and DP3-5; *p* = 0.053) (Figure 3A). Upon combined exposure to DP3-5 and CpG, significantly increased galectin-9 concentrations were observed as compared to CpG alone. Meanwhile for IL-10, this did not reach significance (*p* = 0.06) (Figure 3B,C).

Th1-type IFNγ secretion was increased by GOS DP4-7 when combined with CpG as compared to CpG alone. However, as GOS DP3-5 and CpG enhanced galectin-9 secretion, while showing a similar pattern for IFNγ and IL-10 secretion, the following studies were performed using GOS DP3-5.

Additionally, the GOS DP3-5 fraction was further separated into DP3, DP4, and DP5 fractions to study the most active oligomer/s within GOS DP3-5. Therefore, IEC were apically exposed to 0.5% (*w*/*v*) DP3, DP4, and DP5 in the presence or absence of 5 µM CpG. In the basolateral compartment, αCD3/CD28-activated PBMC were added and incubated for 24 h.

There was no effect on IFNγ, IL-10, and galectin-9 concentrations upon exposure to GOS DP3-5, DP3, DP4, and DP5 in the absence of CpG, except for DP4 and DP5, which significantly increased IFNγ concentrations (Figure 3D–F). CpG alone significantly increased IFNγ and IL-10 concentrations but did not affect galectin-9 secretion (Figure 3D–F). Only exposure to DP3 in combination with CpG further increased IFNγ concentrations compared to CpG alone. DP3-5 did not enhance IFNγ but, similar to DP3, showed a similar pattern in IL-10 and galectin-9 concentrations, although these did not reach significance (Figure 3D–F). No effect was observed on IFNγ, IL-10, and galectin-9 concentrations upon exposure to DP4 and DP5 in combination with CpG (Figure 3D–F).

### 3.2. Galectin-9 mRNA Expression and IEC-Derived Galectin-9 Secretion by Apical Exposure to GOS DP3-5 and CpG

To study the involvement of galectin-9 secretion resulting from apical GOS DP3-5 and 5 µM CpG exposure, IEC were collected after IEC/PBMC co-culture. In addition to this, IEC were washed and incubated in fresh medium for up to 48 h (24 h in IEC/PBMC co-culture and an additional 24 h in culture of IEC in fresh medium), after which galectin-9 mRNA expression and IEC-derived galectin-9 secretion were studied.

**Figure 4:**
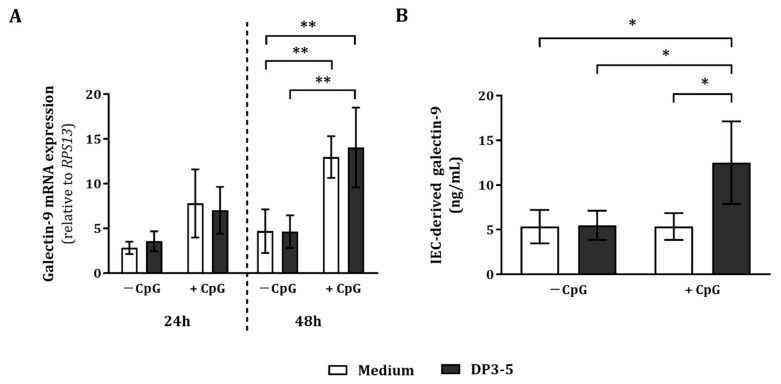
Galectin-9 mRNA expression and IEC-derived galectin-9 release. After IEC/PBMC co-culture, IEC were collected and galectin-9 mRNA expression was measured (**A**). Alternatively, IEC were washed and incubated in fresh medium for an additional 24 h (48 h in total; 24 h in IEC/PBMC co-culture and an additional 24 h in IEC culture in fresh medium), after which IEC and the basolateral supernatant were collected. The relative galectin-9 mRNA abundance at 24 h and 48 h (**A**) and the IEC-derived galectin-9 secretion at 48 h (**B**) were analyzed. Data represent mean ± SEM of *n* = 3 and *n* = 6 independent PBMC donors for (**A**,**B**), respectively (* *p* < 0.05, ** *p* < 0.01).

In IEC, the relative galectin-9 mRNA abundance was not significantly affected by GOS DP3-5, CpG, or the combination after 24 h in IEC/PBMC co-culture (Figure 4A). However, after 48 h (24 h in IEC/PBMC co-culture and an additional 24 h in IEC culture in fresh medium) of exposure to CpG alone in the presence or absence of GOS DP3-5, galectin-9 mRNA expression was significantly increased as compared to the medium control and/or GOS DP3-5 exposure (Figure 4A).

IEC-derived galectin-9 secretion at 48 h was not affected by GOS DP3-5 or CpG alone (Figure 4B). Combined exposure to GOS DP3-5 and CpG significantly increased IEC-derived galectin-9 as compared to the medium, GOS DP3-5, and CpG alone (Figure 4B).

Galectin-9 mRNA expression, but not IEC-derived galectin-9 secretion, was upregulated by CpG. Combined exposure to GOS DP3-5 and CpG upregulated both galectin-9 mRNA expression, as well as IEC-derived galectin-9 secretion.

### 3.3. Increased Th1-Type IFNγ and Regulatory Galectin-9 by β-3′GL and CpG in the IEC/PBMC Co-Culture Model

GOS DP3-5 and GOS DP3 showed immunomodulatory capacities when combined with CpG. Therefore, we aimed to study a specific DP3-sized NDO present in the GOS mixture and found in human milk, namely β-3′GL. IEC were apically exposed to β-3′GL (0.1 and 0.5% *w*/*v*) alone or in combination with 0.1 µM CpG and basolaterally to activated PBMC after which IFNγ, IL-10, and galectin-9 secretion was studied. Additionally, IEC-derived galectins were measured after an additional 24 h of incubation of IEC with fresh medium (48 h in total). Instead of 5 µM CpG, we used 0.1 µM in the following experiments in order to better identify the additional effects of NDO on top of the CpG effect, as was shown for GOS DP3-5 in Figure S1.

**Figure 5:**
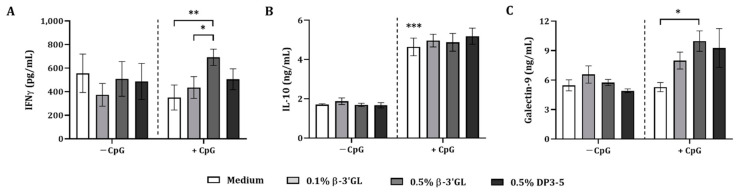
Cytokine and galectin-9 secretion by β-3′GL and CpG in the IEC/PBMC co-culture model. IEC were apically exposed to β-3′GL (0.1% and 0.5% *w*/*v*) or GOS DP3-5 (0.5% *w*/*v*) in combination with 0.1 µM CpG and basolaterally to αCD3/CD28-activated PBMC. After 24 h of incubation, IFNγ (**A**), IL-10 (**B**), and galectin-9 (**C**) were measured in the basolateral supernatant. The data shown are represented as mean ± SEM of *n* = 6 independent PBMC donors (except for IFNγ *n* = 5) (* *p* < 0.05. ** *p* < 0.01, *** *p* < 0.001).

Exposure to β-3′GL, in either concentration, or GOS DP3-5 alone did not have an effect on IFNγ, IL-10, and galectin-9 concentrations (Figure 5A–C). Exposure to CpG alone significantly increased IL-10 concentrations as compared to the medium control but did not affect IFNγ and galectin-9 concentrations (Figure 5A–C). There was no effect on the cytokine and galectin secretion upon combined exposure to CpG and 0.1% β-3′GL or DP3-5 (Figure 5A–C). When IEC were exposed to 0.5% β-3′GL and CpG, significantly increased IFNγ and galectin-9 concentrations were observed, as compared to CpG alone or to 0.1% β-3′GL and CpG (Figure 5A,C). CpG-induced IL-10 concentrations were not further enhanced by 0.5% β-3′GL or GOS DP3-5 (Figure 5B).

Apical exposure of IEC to GOS DP3-5 or β-3′GL alone did not have an effect on the cytokine secretion in the IEC/PBMC co-culture model. Exposure to 0.5% β-3′GL and CpG more strongly promoted the secretion of Th1-type IFNγ and regulatory IL-10 compared to 0.1% β-3′GL or GOS DP3-5 and CpG. For the following studies, 0.5% β-3′GL was used.

### 3.4. Increased Epithelial-Derived Galectin Secretion by β-3′GL and CpG

To further study the immunomodulatory effects of β-3′GL and CpG and the involvement of galectins in the IEC/PBMC co-culture model, IEC were exposed to GOS DP3-5 or β-3′GL (0.5% *w*/*v*) and 0.1 µM CpG.

**Figure 6:**
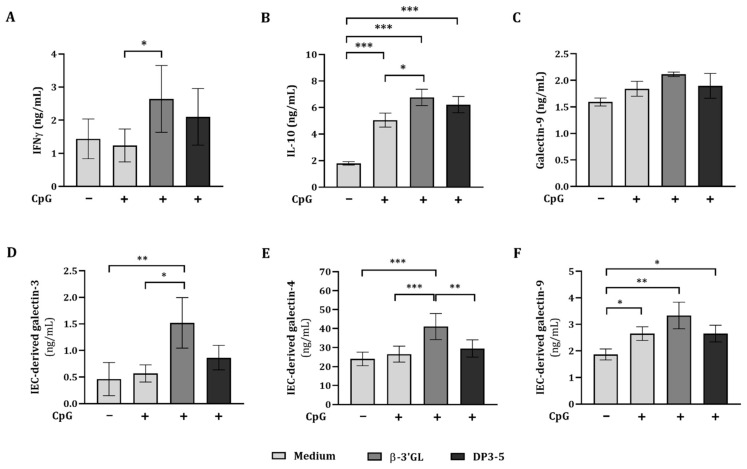
Cytokine and galectin-9 secretion in IEC/PBMC co-culture and IEC-derived galectin secretion at 48 h. IEC were apically exposed to GOS DP3-5 or β-3′GL (0.5% *w*/*v*) in combination with 0.1 µM CpG, and basolaterally to αCD3/CD28-activated PBMC. After 24 h of incubation, IFNγ (**A**), IL-10 (**B**), and galectin-9 (**C**) were measured in the basolateral compartment. Additionally, after IEC/PBMC co-culture, IEC were washed and incubated in fresh medium for an additional 24 h (48 h in total; 24 h in IEC/PBMC culture and an additional 24 h in IEC culture in medium), after which IEC-derived galectin-3 (**D**), -4 (**E**), and -9 (**F**) were measured in the basolateral supernatant. The data shown are represented as mean ± SEM of *n* = 6 independent PBMC donors (except for galectin-9 *n* = 4) (* *p* < 0.05. ** *p* < 0.01, *** *p* < 0.001).

Exposure to CpG alone did not affect IFNγ or galectin-9 concentrations but significantly increased IL-10 concentrations compared to medium control levels (Figure 6A–C). Combined exposure to CpG and β-3′GL or GOS DP3-5 significantly increased IL-10 concentrations as compared to the medium control and/or CpG alone, but no effect was observed on galectin-9 concentrations (Figure 6B,C). Increased IFNγ concentrations were observed upon β-3′GL and CpG as compared to CpG alone (Figure 6A). Significantly decreased IL-13 and TNFα concentrations were observed upon exposure to CpG alone as compared to the medium control (Figure S2). These remained reduced and were not further affected by combined exposure to CpG and GOS DP3-5 or β-3′GL.

To study the involvement of epithelial-derived mediators in the immunomodulatory effects promoted by NDO and CpG, IEC-derived galectin-3, -4, and -9 secretion was measured after 48 h (24 h in IEC/PBMC co-culture and an additional 24 h in IEC culture in fresh medium) and correlated to the cytokine secretion in the IEC/PBMC co-culture. Additionally, galectin-3, -4, and -9 mRNA expression was measured at 48 h.

There was no effect on the IEC-derived galectin-3 and -4 secretion by CpG alone, but increased IEC-derived galectin-9 was observed as compared to the medium control (Figure 6D–F). The increased IEC-derived galectin-9 was also observed upon exposure to GOS DP3-5 or β-3′GL in combination with CpG as compared to the medium control (Figure 6F). Only upon combined exposure to β-3′GL and CpG significant increases were observed in IEC-derived galectin-3 and -4 concentrations as compared to the medium control, CpG alone, and/or GOS DP3-5 and CpG (Figure 6D,E).

In order to link the IEC-derived galectins with the outcome of the immune response, correlations were calculated. These results are summarized in Table 1. A positive correlation was observed between IFNγ and IEC-derived galectin-3, -4, and -9. There was no correlation between IL-10 and IEC-derived galectin-3 and -4. However, a positive correlation was observed between IL-10 and IEC-derived-9. Only IEC-derived galectin-9 was positively correlated to galectin-9, but not to IEC-derived galectin-3 or -4. A negative correlation was observed between IL-13 and IEC-derived galectin-3 and -9, but not with galectin-4 (Figure S2). Meanwhile, TNFα concentrations were not correlated to epithelial-derived galectins (Figure S2).

IEC-derived galectin-3, -4, and -9 were upregulated upon the exposure of IEC to β-3′GL and CpG in the IEC/PBMC co-culture model. IEC-derived galectin-9 secretion was correlated positively to IFNγ, IL-10, and galectin-9 secretion and negatively to IL-13 in the IEC/PBMC co-culture. However, IEC-derived galectin-3 was correlated positively to IFNγ and negatively to IL-13, while IEC-derived galectin-4 was correlated only to IFNγ concentrations.

Additionally, the galectin gene expression was measured at 48 h. There was no effect on the galectin-3, -4, and -9 mRNA expression upon exposure to 0.1 µM CpG alone or in the presence of DP3-5 (Figure S3). Combined exposure to β-3′GL and CpG significantly decreased galectin-4 mRNA expression as compared to the medium control or GOS DP3-5 in combination with CpG (Figure S3). Galectin-9 mRNA expression was significantly increased by exposure to β-3′GL and CpG as compared to GOS DP3-5 and CpG (Figure S3).

## 4. Discussion

Human milk is highly abundant in NDO for which diverse immune regulatory functions have been described [1]. GOS comprise the main component in the prebiotic mixture resembling the amount and structure diversity of NDO in human milk [2,4] and are composed of a complex variety of NDO with DPs ranging between 2 and 8 [14]. The purpose of this study was to evaluate the most active oligomers within the GOS mixture regarding the immunomodulatory capacity using the IEC/PBMC co-culture model in which the crosstalk between IEC and immune cells was studied.

Up to 60% of GOS DP structures have a DP size of DP2 or DP3 [13,14]. Within the DP2 fraction, various NDO structures have been characterized also including lactose residues [14]. Previous studies did not observe immunomodulatory effects upon exposure to lactose in the IEC/PBMC co-culture model [35]. However, due to the presence of lactose and the inability to separate this from other possible active NDO within the DP2 fraction, this study focused on analyzing GOS fractions with size of DP3 and longer fractions, namely DP4-7, DP3-7, and DP3-5 fractions, which were isolated by size-exclusion chromatography.

In the current study, Th1-type IFNγ concentrations were increased upon exposure to GOS DP4-7 in association with CpG. However, only GOS DP3-5 showed increased CpG-induced regulatory-type galectin-9 secretion with a similar Th1 secretion, suggesting a pattern of Th1 and regulatory cytokine secretion. Therefore, GOS DP3-5 was used for further studies, based on previous findings identifying galectin-9 as a key mediator in driving a regulatory-type Th1 response [31,33,36]. The current study showed the presence of structures with immunomodulatory properties within the GOS DP3-5 fraction, and, also within this fraction, GOS DP3 was identified for being able to upregulate IFNγ concentrations only when combined with CpG. This suggests that the most active oligomers in terms of Th1-type regulatory immunomodulation might be DP3-sized.

Previous studies investigated the involvement of galectins in general and galectin-9 in particular in supporting the regulatory-type Th1 immunomodulatory effects by the blocking of galectins, which resulted in the suppression of regulatory-type Th1 immune effects [31]. Furthermore, stimulation of αCD3/CD28-activated PBMC with recombinant galectin-9 enhanced IFNγ and IL-10 secretion and increased the percentage of Th1 and regulatory T-cells [10], which reinforces the role of galectin-9 as a key immune regulator.

The TLR9 agonist CpG used to mimic a bacterial trigger (bacterial DNA) in the IEC/PBMC co-culture model was required to support the immunomodulatory effects of NDO. Although TLR9 is mostly described as an endosomal receptor, previous studies have observed a surface expression of TLR9 in IEC [32,38,39,40]. Even though combined exposure to CpG and GOS DP3-5 resulted in increased galectin-9 secretion, the addition of GOS DP3-5 did not result in the further upregulation in galectin-9 mRNA in the IEC at 24 h or at 48 h after apical CpG exposure. This suggests that CpG can regulate galectin-9 expression at the level of gene transcription, while the oligosaccharides facilitate the basolateral release of galectin-9, and thereby support mucosal immunomodulation and/or development.

**Figure 7:**
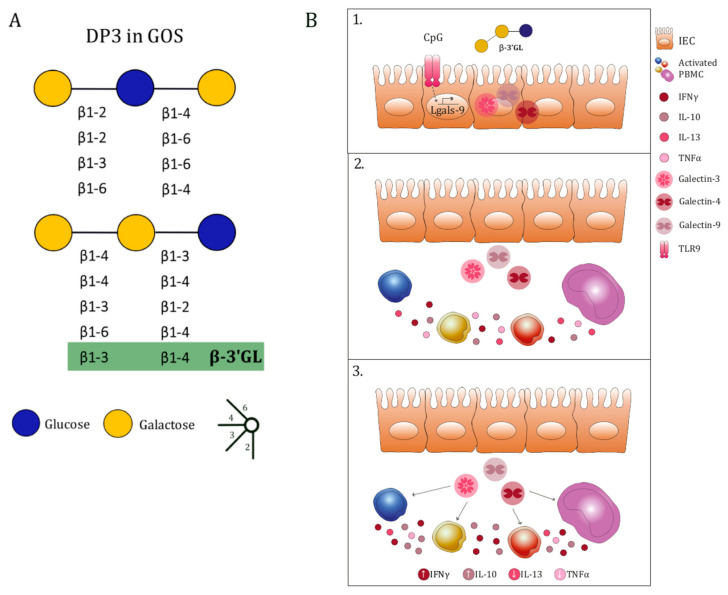
DP3-sized oligomers in GOS and description of the effects by β-3′GL and CpG in the IEC/PBMC co-culture model. DP3-sized structures present in GOS including β-3′GL are shown in (**A**). A summary of the effects observed in the IEC/PBMC co-culture model upon combined exposure to β-3′GL and CpG is shown in (**B**). Galectins are synthesized in the cytosol of IEC upon exposure to β-3′GL and CpG. Additionally, an upregulation of galectin-9 mRNA expression might be promoted by CpG (1). Galectins are then pushed out from the IEC toward the underlying immune compartment (2). Upon inflammatory conditions, defined by the activation of PBMC using αCD3/CD28, the immune cells in the basolateral compartment representing the lamina propria produce cytokines (2). Combined exposure to β-3′GL and CpG enhances IEC-derived galectin-3, -4, and -9 secretion. The IEC-derived galectins modulate the cytokine secretion by upregulating IFNγ and IL-10 while downregulating IL-13 and TNFα secretion (3).

As GOS DP3-5 showed the most potent immunomodulatory activity out of the DP fractions tested, and this effect was mimicked to some extent by GOS DP3, we further investigated the capacity of a DP3-sized NDO, namely β-3′GL, in promoting immunomodulatory effects. A recent study determined the presence of low concentrations of β-3′GL in human milk samples [36]. Furthermore, β-3′GL is present in the GOS DP3 mixture (Figure 7A) [14]. Thus, we further studied the structure-specific effects of β-3′GL compared to GOS DP3-5 in combination with CpG in the IEC/PBMC model. Increasing the concentration of β-3′GL to 0.5% (*w*/*v*) supported a Th1-type regulatory immune response, as shown by increased IFNγ and galectin-9 or IL-10 secretion on top of CpG alone, which is in line with previous studies describing the immunomodulatory effects of other NDO [29,30,31,34]. In those studies, similar immune polarization profiles for GOS/FOS and 2′fucosyllactose (2′FL), a NDO abundantly present in human milk, were also shown in the presence of CpG [29,31], suggesting that this type of immunomodulation may be relevant for immune development [1]. Similar to 2′FL, β-3′GL may be an important NDO structure present in human milk, capable of supporting mucosal immune development driven by microbial signals (such as bacterial CpG DNA) in early life [29].

Due to the influence of IEC-derived galectins in supporting the immunomodulatory effects boosted by NDO and CpG described in previous studies [29,30,31,34], we studied IEC-derived galectin secretion. Only β-3′GL in the presence of CpG boosted the secretion of IEC-derived galectin-3, -4, and -9. However, only IEC-derived galectin-9 secretion was upregulated by DP3-5 and CpG. Besides, only IEC-derived galectin-9, but not IEC-derived galectin-3 or -4, correlated to IL-10 and galectin-9 secretion in the IEC/PBMC co-culture, which indicates that IEC-derived galectin-9 supports the regulatory-type immunity. Other studies have confirmed that incubation of activated PBMC with recombinant human galectin-9 was able to enhance not only IFN-γ secretion, but also regulatory IL-10 release [10]. However, beyond galectin-9, epithelial-derived galectin-3 and -4 were also found to significantly correlate positively with IFNγ concentrations. This suggests that all IEC-derived galectins might have been involved in increasing the IFNγ release by the activated PBMC under β-3′GL and CpG-exposed conditions. These results emphasize the need to better understand the complexity involved within the mucosal interactions between IEC and immune cells, as well as the role of galectins in these processes.

The IEC/PBMC model used was set-up to mimic the epithelial cell and immune cell crosstalk representing the intestinal mucosa. The model makes use of PBMC instead of lamina propria mononuclear cells (LPMC), because LPMC isolation requires access to clinical bowel samples and a laborious isolation procedure [41]. LPMC do show similarities as well as differences in the composition and function when compared to PBMC [42,43]. However, mitogen stimulation or activation via the T-cell receptor in both PBMC and LPMC results in induced levels of IFNγ, IL-10, and TNFα [41,42,43]. Furthermore, the results obtained from the IEC/PBMC model used were validated using in vivo animal models for food allergy [10,11], and in clinical samples of a NDO dietary intervention study, serum galectin-9 levels were shown to be enhanced [10].

The high dose of CpG was found to enhance the transcription of galectin-9; however, this was not further modified by GOS DP3-5, while it was capable of enhancing galectin-9 secretion. In addition, the effects of β-3′GL on epithelial galectin release could not be explained by increased galectin mRNA expression. Little is known about the factors inducing the transcription of galectins, as well as their intracellular storage. Galectins are widely known for their ability to recognize and bind extracellular carbohydrates with high affinity for β-galactoside structures such as NDO. Several studies have described the affinity of specific NDO structures to galectins according to their chemical structure and observed how chemical modifications such as fucosylation or galactosylation, among others, can improve the binding affinity of galectins to specific NDO structures. The increased binding affinity might, in turn, result in improved biological functions of NDO such as raft formation or attachment to pathogenic bacteria [44,45,46,47]. Although further research is needed, recent studies have proposed that changes in O-GlcNAcylation might be involved in regulating galectin expression [48].

To summarize, GOS DP3-5 in the presence of CpG, a TLR9 agonist mimicking a bacterial trigger, was able to promote immunomodulatory effects by enhancing immune responses in association with modified epithelial-derived galectin secretion. Moreover, exposure to DP3-sized β-3′GL, a NDO present in human milk, was most effective in enhancing CpG-induced galectin-9 release, while also enhancing galectin-3 and -4 secretion, which correlated with the instruction of a regulatory-type Th1 response. The most relevant findings described in this manuscript are summarized in Figure 7B. The use of PBMC might not fully resemble the immune cell populations present in the lamina propria and, therefore, the immune responses might differ from those shown in this manuscript. However, previous studies have shown the predictive value of the IEC/PBMC model in vivo in a murine model for food allergy, by identifying dietary interventions with immunomodulatory properties in which galectin-9 had a key role [10]. This emphasizes the translational value of the IEC/PBMC co-culture model. Our aim is to use the IEC/PBMC co-culture model as a first step to identify relevant bioactive components that should later be studied using more complex models. In addition to this, by the use of these models combining epithelial and immune cells, we aim to better understand the complex interactions occurring between these types of cells, which further contribute to our understanding.

In conclusion, epithelial-derived galectins were demonstrated to be key players in the mucosal immune development supported by GOS of which DP3-size β-3′GL showed relevant immunomodulatory properties.

## Figures and Tables

**Table 1:** Correlations of cytokines and galectin-9 in the IEC/PBMC co-culture and IEC-derived galectins. Cytokine and galectin-9 secretions measured in the IEC/PBMC co-culture were correlated to IEC-derived galectin concentrations (48 h) using Pearson correlations.

	IEC-Derived Galectin-3	IEC-Derived Galectin-4	IEC-Derived Galectin-9
IFNγ	+	+	+
	r = 0.65	r = 0.49	r = 0.38
	*p* < 0.0001	*p* = 0.003	*p* = 0.02
IL-10	n.s.	n.s.	+
	r = 0.06	r = 0.07	r = 0.37
	*p* = 0.7	*p* = 0.7	*p* = 0.03
Galectin-9	n.s.	n.s.	+
	r = 0.02	r = 0.3	r = 0.44
	*p* = 0.9	*p* = 0.1	*p* = 0.03

+ Positive correlation; n.s. nonsignificant correlation.

## Data Availability

The data presented in this study are available upon request from the corresponding author.

## Supplementary Materials

The following supporting information can be downloaded at: https://www.mdpi.com/article/10.3390/biom12030384/s1, Figure S1: Lower CpG concentrations effectively supported immunomodulatory effects by GOS DP3-5; Figure S2: Cytokine secretion in IEC/PBMC co-culture model; Figure S3: Galectin mRNA expression.

## Abbreviations

β-3′galactosyllactose (β-3′GL); 2′fucosyllactose (2′FL); complementary DNA (cDNA), electrospray ionization-mass spectrometry (ESI-MS); enzyme-linked immunosorbent assay (ELISA); fetal calf serum (FCS); fructo-oligosaccharides (FOS); galacto-oligosaccharides (GOS); intestinal epithelial cells (IEC); lamina propria mononuclear cells (LPMC); non-digestible oligosaccharides (NDO); oligodeoxynucleotides (ODN); peripheral blood mononuclear cells (PBMC); quantitative Polymerase Chain Reaction (qPCR).
