# Supplementary material for: Modulation of the Epithelial-Immune Cell Crosstalk and Related Galectin Secretion by DP3-5 Galacto-Oligosaccharides and β-3′Galactosyllactose
Source: Biomolecules. 2022 Feb 28;12(3):384. doi: 10.3390/biom12030384 (PMC8945669; doi:10.3390/biom12030384)
Supplement: Supplementary file 1 [file biomolecules-12-00384-s001.zip › biomolecules-1561046-supplementary.pdf]

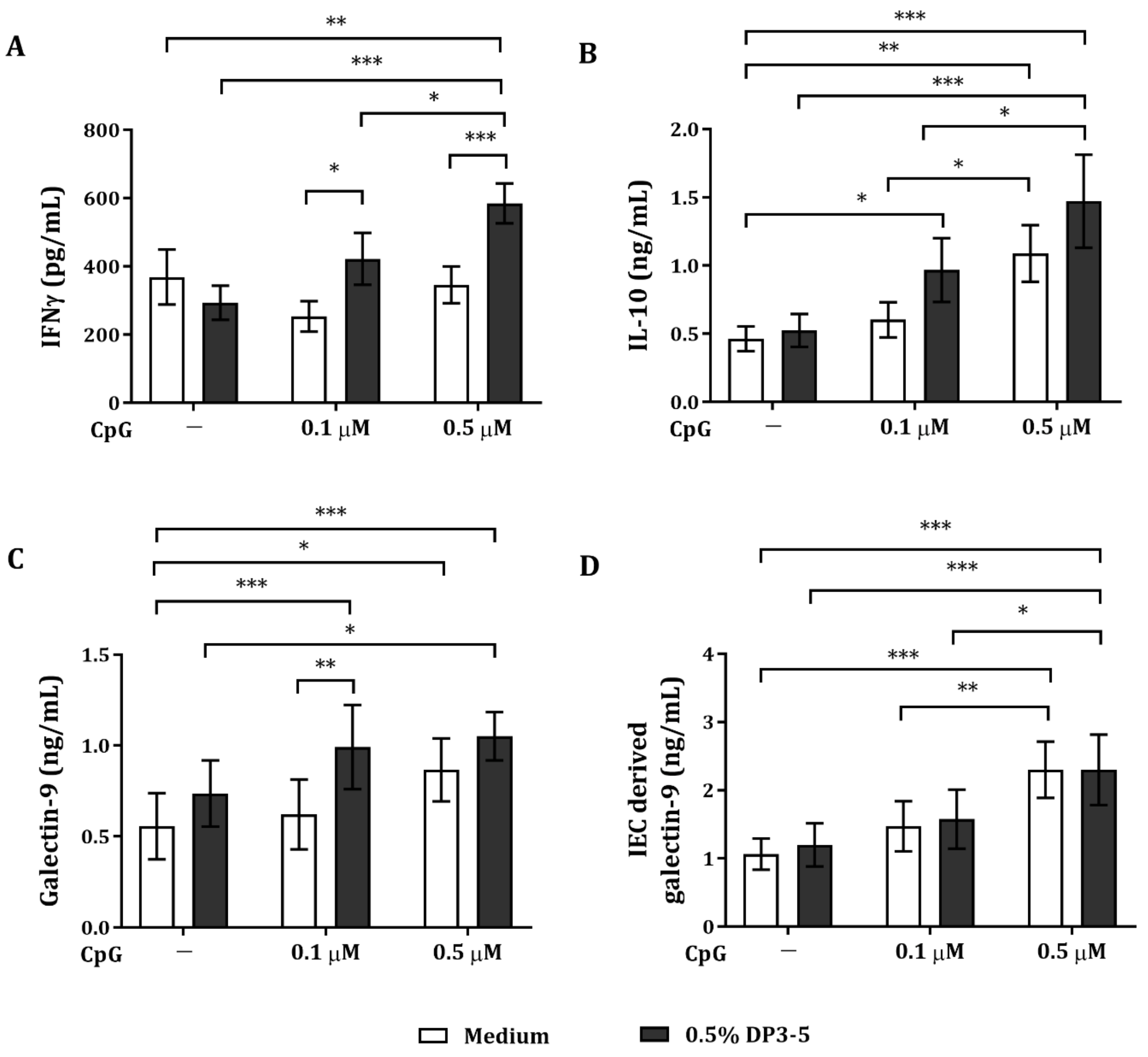

Figure S1. Lower CpG concentrations effectively supported immunomodulatory effects by GOS DP3-5. IEC were apically exposed to 0.5% GOS DP3-5 (*w/v*) in combination with 0.1 or 0.5 μM CpG and basolaterally to αCD3/CD28-activated PBMC. After 24 hours incubation, IFNγ (A), IL-10 (B) and galectin-9 (C) were measured in the basolateral supernatant. After IEC/PBMC co-culture, IEC were washed and incubated in fresh medium for additional 24 hours after which IEC-derived galectin-9 (D) was measured. Data represent mean ± SEM of n = 6 independent PBMC donors (n = 5 for IFNγ) (* $p < 0.05$. ** $p < 0.01$, *** $p < 0.001$).

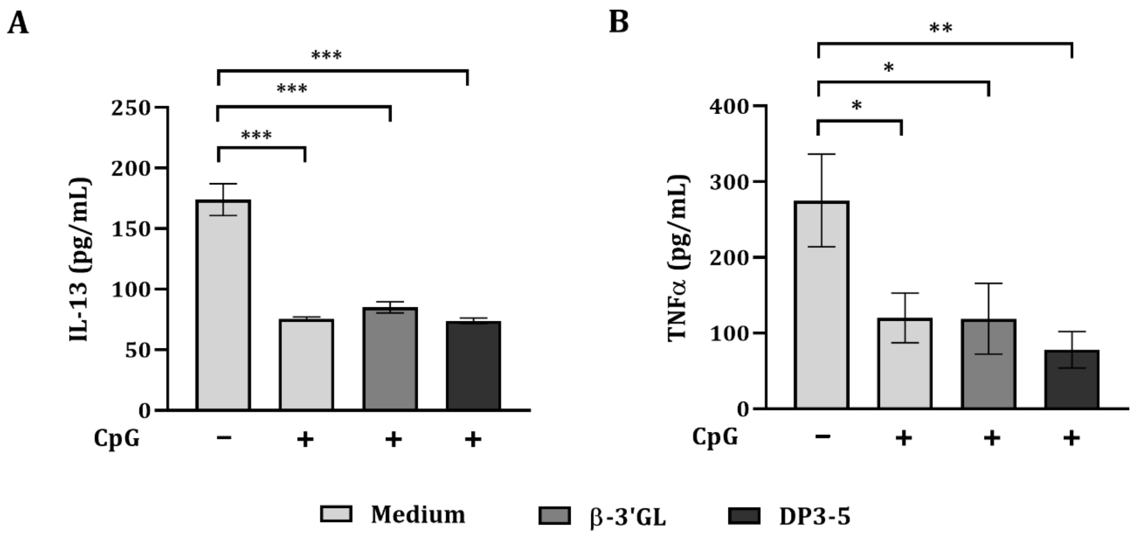

	IEC-derived galectin-3	IEC-derived galectin-4	IEC-derived galectin-9
TNFα	n.s.	n.s.	n.s.
	r = 0.32	r = 0.32	r = – 0.009
	p = 0.06	p = 0.06	p = 0.96
IL-13	–	n.s.	–
	r = – 0.42	r = – 0.34	r = – 0.54
	p = 0.04	p = 0.1	p = 0.007

– Negative correlation; n.s. non-significant correlation

Figure S2. Cytokine secretion in IEC/PBMC co-culture model. IEC were apically exposed to GOS DP3-5 or β-3'GL (0.5% *w/v*) in combination with 0.1 μM CpG, and basolaterally to αCD3/CD28-activated PBMC. After 24 hours incubation, TNFα (A) and IL-13 (B) were measured in the basolateral compartment. The data shown are represented as mean ± SEM of $n = 4$ independent PBMC donors for IL-13 and $n = 6$ for TNFα. Additionally, IL-13 and TNFα secretion was correlated using Pearson correlation to IEC-derived galectins as shown in table (C) (* $p < 0.05$. ** $p < 0.01$, *** $p < 0.001$). Negative correlations are represented as (–) and (n.s.) represents non-significant correlations.

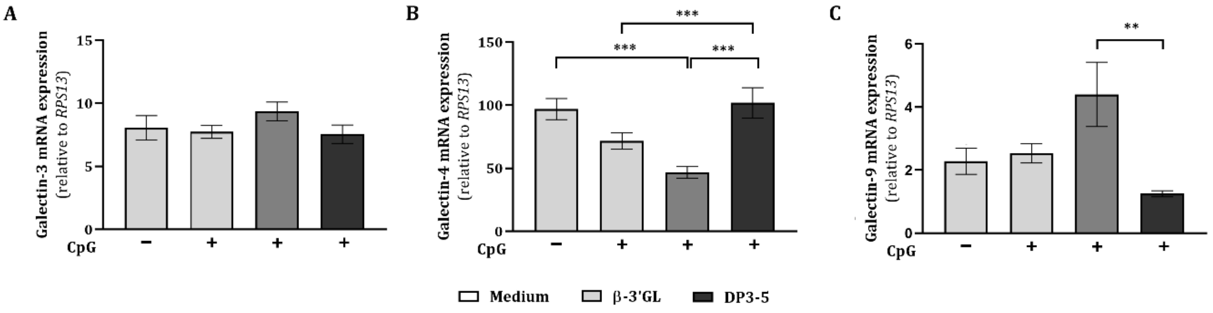

Figure S3. Galectin mRNA expression. After IEC/PBMC co-culture, IEC were separated from the PBMC fraction, washed and cultured in fresh medium for 24 hours after which IEC were collected and the relative mRNA expression of galectin-3 (A), -4 (B) and -9 (C) was measured. Data represent mean ± SEM of n = 6 independent PBMC donors (** $p < 0.01$, *** $p < 0.001$).
